# Swipe, click, regret: an opinion on persuasive e-commerce and consumer autonomy

**DOI:** 10.3389/frai.2025.1684841

**Published:** 2025-10-20

**Authors:** Alain Monica George, Rupa R

**Affiliations:** Department of Commerce, Marian College Kuttikkanam Autonomous, Kerala, India

**Keywords:** persuasive e-commerce, consumer autonomy, impulse buying, behavioral economics, digital nudging, buy now pay later (BNPL), online shopping behavior

## 1 Introduction

Over the past 20 years, e-commerce has fully transitioned from being a secondary channel of shopping to an embedded aspect of daily life in the marketplace. The expansion of e-commerce is driven by technological advancements, as well as intentional choices of design that prioritize engagement and conversion. Online shopping is not a digital version of retail; instead, it provides an enduring and interactive digital environment that influences consumer decisions, shaped by behavioral research, persuasive design, and AI-enabled customization.

For example, Amazon, Flipkart, and social commerce exhibit the manifestations of this evolution. “We only have 1 left!” Personal recommendations, countdown timers, and notifications are indicative of what Thaler and Sunstein (2008) refer to as recounted “choice architecture”—choice environments established specifically to steer behavior. While these design features are marketed as convenience, they provide a space to discourage thinking and ultimately promote impulse buying, which connects to Rook's (1987) earlier view of impulsive choice.

Similarly, gamification—not just in the form of gamification in the typical video-game-styled engagement with loyalty points, limited-time quests, and interactive product reveals—places even greater emphasis on the crossover of entertainment and consumption. Social commerce, along with branded influencer marketing, further integrates retail and everyday interaction, which creates the ongoing exposure to subtle nudges to spend. Behind these behaviors are teams of behavioral scientists, user experience (UX) designers, artificial intelligence (AI) engineers, and marketers all optimizing consumer micro-decisions. Thus, where does personalization transition to manipulation, and how much agency does the consumer have when attention is the currency in a marketplace?

The impact extends beyond financial repercussions to psychological sequelae such as post-purchase regret, debt cycles, and compulsive buying. Mechanisms like Buy Now, Pay Later (BNPL) programs exacerbate these risks, with the separation of consideration from consequence heightening risk. This article analyses persuasive e-commerce processes via behavioral economics and consumer psychology, suggesting that contemporary design practices focus on platform profitability and disregard consumer autonomy. We offer a call for reflective design alongside regulations that reintroduce deliberation and protect consumers in online marketplaces.

## 2 Methodology

This article presents a conceptual commentary based on opinion examining how persuasion in e-commerce can shape consumer decision-making and impulsive buying behaviors. It employs a desk-based approach based on secondary sourcing from published academic literature, industry reports, and behaviors observed from platform practices. The approach taken in the article is also interdisciplinary, using perspectives from behavioral economics, consumer psychology, UX design, and AI-driven personalization, alongside regulatory perspectives.

We discuss established theories, drawing from Thaler and Sunstein's Nudge Theory (Thaler and Sunstein, 2008), Rook's (1987) work on impulse buying, and Verplanken and Herabadi's (2001) Impulse Buying Tendency. We locate those theories of consumer behavior in AI-enabled digital e-commerce settings containing nudges and manipulative clearance. “We do not strive for generalizability, as this is a commentary interpretation, not an empirical research paper, although we do strive for reflexivity. We share practical examples from platforms to illustrate a case for ethical and regulatory considerations to be thoughtfully considered for when examining autonomy for consumers in these designed-for contexts.

## 3 Theoretical framework: nudging, impulse, and digital choice

In this commentary, we will examine the persuasive e-commerce “swipe, click, regret” loop from three angles. Thaler and Sunstein's Nudge Theory (2008) first shows how signifiers of design—scarcity alerts (limited stock), countdown timers (X hours left to buy), and recommending products (X of your friends bought)—influence consumer decision-making, moving them toward fast responses, while simultaneously allowing the consumer to feel as if they are making a choice. Second, Rook (1987) defines impulse buying as affecting the individual emotionally and considered it an action indicative of deterioration in deliberation. Finally, Verplanken and Herabadi's (2001) Impulse Buying Tendency model characterizes impulse buying as stable tendencies that can be influenced by digital platforms. Together, these theories position how personalization and gamification can enhance the construct of impulsivity. E-commerce platforms intentionally construct digital architectures that invoke behavioral heuristics and bias and turn autonomy into exploitation and convenience into regret (Bergram et al., 2022).

## 4 Current state of persuasive e-commerce

E-commerce platforms have transitioned from sites for buying to designers of situations designed to encourage impulsivity, leveraging credit for consumption. Where Rook (1987) and Thaler and Sunstein (2008) framed impulse and nudging as something one does intermittently or harmlessly, we now want to suggest that it's institutionalized (Oinas-Kukkonen and Harjumaa, 2009). Scarcity signals, urgency messages, and AI personalization now seek to exploit the vulnerabilities of consumers (Singh et al., 2024; Hettler et al., 2024; Gupta, 2025).

Buy now, pay later (BNPL) is a prime example of this shift. Research suggests that BNPL use is associated with impulse buying, materialistic tendencies, and a lack of self-control in both Gen Z and Millennials (Widayati et al., 2024; Raj et al., 2023; Aisjah, 2024). Ecological studies show that BNPL leads to overdraft and debt, particularly among low-income users (DeHaan et al., 2024; O'Brien et al., 2024; Raj et al., 2024), suggesting that the persuasive commerce has shifted from a simple aid in habitual consumer behavior to one that takes advantage of behavioral bias (Bhatia et al., 2022).

## 5 Emotional and behavioral consequences

Persuasive e-commerce can be defined as shopping simplified into a reflex: swipe, click, and regret. The pleasure of instant gratification will always run downhill to anxiety, guilt, or shame. Most consumers will not stop there and will return to the shopping platform to again engage in the act of seeking purchase to fill the relief void, thus creating a compulsive loop. Unlike traditional shopping methods, devices such as one-click checkout and Buy Now, Pay Later (BNPL) eliminate the sacred pause time for contemplation and disconnect payment from consequence (Lupşa-Tătaru et al., 2023).

This design does not merely reduce friction or discomfort; it weaponizes friction (De Abreu Lessa, 2024). It's the platforms that cash in on repeated lapses of self-control while the consumers “own” the emotional toll and eventually, the long-term financial toll (Griffiths, 2005). Convenience in this space enables manipulation.

There is a lack of consistency in how regulators respond to various forms of persuasive commerce (Anwar Baig, 2024). In the UK, Australia, and New Zealand, buy now pay later is classified as credit, meaning the laws require an affordability check and notices. The EU has established more protections with the 2021 Consumer Credit Directive and Directive 2019/2161, requiring, among other things, more transparency for online reviews and penalties for misleading practices (Đurović, 2020). Most recently, India's Consumer Protection Act 2019 is relevant as it established the Central Consumer Protection Authority and e-commerce rules prohibiting dark patterns, false scarcity cues, and manipulative defaults (Sao, 2025; Ayilyath, 2020). These measures address the swipe, click, and regret cycle by limiting exploitative designs that elicit impulsive purchases and monetize consumer autonomy.

Nonetheless, enforcement still struggles to keep pace with technological developments on the part of the platforms. AI engineers and behavioral teams are faster than regulators and can exploit divergent jurisdictions to build persuasive designs (Meškić et al., 2022; Wibowo, 2024). Unless we have a global, cohesive, and enforceable standard created based on the OECD or UNCTAD processes, this form of commerce is likely to become further entrenched—showing manipulation as convenience—leaving consumers even more vulnerable going forward.

## 6 Toward reflective design

Resolving these challenges entails moving from manipulation to reflection. Ultimately, we propose three nudges: (1) pause nudges, (2) transparency nudges, and (3) opt-in personalization, which may provide a basis for reintroducing reflection without sacrificing convenience. While the bulk of evidence is identified in health and education (Mirbabaie et al., 2022; Michels et al., 2022), we also argue that e-commerce is clearly in need of something similar next.

Pause nudges could intervene on a one-click buy now pay later (BNPL) signup; transparency nudges would help identify why a consumer was targeted in the first place (Gupta, 2025); and opt-in personalization would return some agency with what the customer prevails over while purchasing (Maxwell Nwanna et al., 2025). Of course, these are just hypothetical examples and need trials to establish feasibility (Singh et al., 2024; Hettler et al., 2024) but considering the impact manipulatively designed products have had on consumers, we believe a reflective design process should be recognized as an ethical normative reference of some kind for addressing any regulation of product design, digital environments, or innovations (Okesiji et al., 2024).

## 7 Discussion

The implications of persuasive design and AI-mediated personalization design are leading consumers to often forfeit their autonomy. In addition to making credit more accessible, buy now, pay later (BNPL) options tie debt to impulsivity, facilitating debt spirals that people are often unlikely to escape (Syam Kumar and Nayak, 2024; DeHaan et al., 2024). BNPL options are intended to normalize immediate gratification and obscure long-term costs to the point that consumers are swiping and clicking without even stopping to reflect, followed by remorse. Just like adaptive personalization can produce some level of manipulation with the absence of transparency or compulsion of a biased algorithm (Shemshaki, 2024), it is easy for consumers to be nudged, even at a cost to the consumer, to a transaction that may benefit the platform (Aggarwal, 2024; Bitra, 2025).

Indeed, regulatory regimes are now beginning to understand and generate best practices or policies to respond to any one of the weaknesses mentioned above. The EU Directive 2019/2161 effectively has a mandate for transparency with respect to online reviews and some inchoate penalties against deceptive practices. The Consumer Protection Act 2019 in India prohibits dark patterns and false urgency, in addition to empowering the Central Consumer Protection Authority to step in as needed (Đurović, 2020; Sao, 2025). OECD principles certainly similarly outline the same challenges in a global context, again pointing to fairness, accountability, and transparency (Mishra and Varshney, 2024).

However, these attempts at regulation are also generally fragmented (in intentionality and lack of enforcement), and platforms consistently take advantage. They do this by being transjurisdictional (even transnational), moving much faster than the regulation or public policies that govern these platforms (Wibowo, 2024).

## 8 Scope for further research

Future research has many exciting possibilities to investigate the mechanisms and consequences of persuasive e-commerce. Areas of investigation could include the long-term outcomes, in terms of both psychology and finances, of artificial-intelligence-enabled personalization and digital nudging, particularly for compulsive buying and post-purchase regret. Experimental investigations might be employed to examine reflective design interventions (e.g., pause nudges, transparency nudges, opt-in personalization) to see if they support consumer agency without sacrificing usability.

Demographic and socio-economic predictions might lead to studies in which researchers examine cross-cultural differences in susceptibility to persuasive tactics, while structural factors could shape consumers' behaviors on the platform. A comparative policy research agenda comparing the effects of BNPL (buy-now, pay-later) and other consumer protection laws could develop a body of evidence that enables understanding behaviors across contexts and transferability of practices to international systems.

Lastly, vulnerable consumers targeted and profiled when using artificial intelligence will merit deeper analysis, especially regarding data ethics, consent, and algorithmic bias. Addressing the questions above would support developing a more robust body of evidence toward policy intervention and design of digital commerce systems that enable innovation while considering consumers' welfare.
